# Characterization of Bioactive Phenolics and Antioxidant Capacity of Edible Bean Extracts of 50 Fabaceae Populations Grown in Thailand

**DOI:** 10.3390/foods10123118

**Published:** 2021-12-16

**Authors:** Duangjai Tungmunnithum, Samantha Drouet, Jose Manuel Lorenzo, Christophe Hano

**Affiliations:** 1 Department of Pharmaceutical Botany, Faculty of Pharmacy, Mahidol University, Bangkok 10400, Thailand; 2 Laboratoire de Biologie des Ligneux et des Grandes Cultures, Campus Eure et Loir, Orleans University, 28000 Chartres, France; samantha.drouet@univ-orleans.fr; 3 Centro Tecnológico de la Carne de Galicia, Adva. Galicia n° 4, Parque Tecnológico de Galicia, San Cibrao das Viñas, 32900 Ourense, Spain; jmlorenzo@ceteca.net; 4 Área de Tecnología de los Alimentos, Facultad de Ciencias de Ourense, Universidad de Vigo, 32004 Ourense, Spain

**Keywords:** Fabaceae, bioactive phenolics, antioxidant capacity, edible bean, population level

## Abstract

Fabaceae is the third largest family containing great variation among populations. However, previous studies mainly focus on single species, and phytochemicals at population level have never been reported. This work aims to complete this knowledge with 50 populations from throughout Thailand by (1) determining total phenolic (TPC), flavonoid (TFC), and anthocyanin (TAC) contents; and (2) investigating in vitro and cellular antioxidant potentials. Phytochemicals of 50 populations from different localities are differed, illustrating high heterogeneity occurring in polyphenols accumulations. *Vigna unguiculata* subsp. *sesquipedalis* populations showed low variability in TPC ranging from 628.3 to 717.3 mg/100 g DW gallic acid equivalent, whereas the high variability found in TFC and TAC range from 786.9 to 1536.1 mg/100 g DW quercetin equivalent, and 13.4 to 41.6 mg/100 g DW cyanidin equivalent. Red cultivar population #16 had the greatest TAC, but surprisingly the cream cultivars were relatively high in anthocyanins. HPLC quantification of genistein and daidzein showed great variations among populations. In vitro antioxidant results indicated that antioxidant capacity mediated by electron transfer. Cellular antioxidants ranged from 59.7% to 87.9% of ROS/RNS in yeast model. This study investigated at the population level contributing to better and frontier knowledge for nutraceutical/phytopharmaceutical sectors to seek potential raw plant material.

## 1. Introduction

Fabaceae, or the so-called Leguminosae, is the third largest plant family which consists of more than 19,000 species distributed worldwide [1]. This family consists of terrestrial flowering plants, which usually have compound leave with 3-foliolate or 4-foliolate, raceme inflorescences with actinomorphic or bilaterally symmetrical flowers. The fruits of Fabaceae plants usually contain one- to many-seeded legumes, which are dehiscent or indehiscent, dry, or fleshy, inflated or compressed. The species members of Fabaceae have been used in various proposes ranging from food, cosmetics, timber, ornamentals, and medicines to use as fodder and green manure [2,3,4,5,6]. In Thailand, the edible Fabaceae species is one of the most important foods, which have long been consumed, and which are used more than other plant groups [4,7,8].

A large number of Fabaceae species are the important economic plant crops of the world. This plant family is the second most important economic crop plant, following Poaceae, the rice family [9]. Many species of the Fabaceae family consist of edible parts, especially the seeds, which are widely consume as vegetable and food ingredients. Due to the wide distribution of this plant family, there are many reports on benefits of the edible species against various types of chronic diseases i.e., cardiovascular diseases, breast cancer, and cataract development [10,11]. In addition, the bioactive ingredients, particularly phenolic phytochemical compounds, from these edible Fabaceae species are of interest to researchers from nutraceutical and related fields for investigation of their nutraceutical or other novel applications in food [10,11,12,13,14].

Legume seeds are high in proteins, minerals, vitamins, and bioactive substances, making them a central nutrient for the human diet [15]. Legumes are a rich source of bioactive phenolic compounds, which are involved in a variety of physiological and metabolic processes in relation with human health [16]. The seeds contain the majority of the phenolics in legume [16,17,18,19,20,21]. The principal phenolic chemicals found in legume seeds include phenolic acids, (iso)flavonoids, and anthocyanins. These phytochemicals are distributed differently in the seed, with the seed coat being rich in flavonoids, whereas the cotyledon mostly contains non-flavonoids, such hydroxycinnamic and hydroxybenzoic acids [22]. Seed legumes are appealing candidates for producing novel functional foods, since they constitute an essential source of these phytochemicals with high antioxidant potential, the ability to scavenge free radicals, and the ability to interact with proteins [16,17]. In addition to this potent antioxidant action, anticarcinogenic, anti-thrombotic, anti-ulcer, anti-atherogenic, anti-allergenic, anti-inflammatory, immunomodulating, anti-microbial, cardioprotective, vasodilatory, and analgesic compounds are just some of the described health benefits [16,17]. Isoflavones are phenolic compounds that have a similar molecular structure to estradiol [17]. In human nutrition, legume-derived products are the most important source of isoflavones. The most physiologically active isoflavones candidates for human health promotion are genistein and daidzein [18,19,20,21].

The antioxidant activity of all these phenolic compounds accumulated in legume seeds has been thoroughly documented, and it is commonly accepted that it is related to their amount or chemical structures, such as the position of hydroxyl groups. The majority of knowledge on antioxidant activity in legumes, however, is based on a small number of species or cultivars that have been examined so far [23]. Furthermore, antioxidant activity is typically assessed using a limited number of assays most of the time limited to in vitro assays. The antioxidant activity of plant extracts cannot be assessed using a single method due to the complex nature of phytochemicals and, in particular, since the assessment of antioxidant activity is largely dependent on the reaction mechanism involved [24,25]. Furthermore, the extraction methods of phenolic compounds from legume seed varies widely in published studies, making it difficult, if not impossible, to evaluate whether or not changes in antioxidant activity reported for distinct legume species occur [23]. Moreover, environmental and agricultural factors, such as location (i.e., soil conditions) and climate, have been demonstrated to have a considerable impact on the accumulation of phenolic compounds and the antioxidant activity [25,26]. To date, there has been no study dealing with the variability in phenolic compounds (phenolics, flavonoids, including the main bioactive isoflavones daidzein and genistein, and anthocyanins) and antioxidant activities determined by a variety of tests able to account for this biological activity for legume seeds from Thai natural populations.

The objective of this study is to complete this knowledge, with 50 populations originating from all Thai regions, by determining the total contents of phenolic, flavonoid (including HPLC determination of daidzein and genistein), and anthocyanin, as well as antioxidant activity determined using three in vitro assays based on different mechanisms and a cellular antioxidant assay.

## 2. Materials and Methods

### 2.1. Chemicals and Reagents

The extraction solvents used in this study were of analytical grade (Thermo Scientific, Illkirch, France). All the reagents for antioxidant tests and standards were provided by Merck (Saint-Quentin Fallavier, France).

### 2.2. Plant Materials

The seed of the 50 Fabaceae populations were sought and collected from all floristic regions in Thailand such as the northern (N), north-eastern (NE), eastern (E), central (C), south-eastern (SE), south-western (SW), and peninsula (PEN) regions. After the literature review and the study of information on the herbarium specimen of Fabaceae plants, the targeted populations in various localities throughout Thailand were sought to find plant materials left in the fields. The collected samples were identified into the species level using the taxonomic key and description in the existing Floras [27,28,29,30], as well as compared with the herbarium specimens kept in Forest Herbarium (BKF), and the Prof. Kasin Suvatabandhu from Herbarium, Chulalongkorn University, (BCU). Herbarium abbreviations are used according to Thiers [31]. Then, the seeds from 50 Fabaceae populations were air-dried, and prepared following the World Health Organization [32] recommendations.

### 2.3. Extraction

Ultrasound-assisted extraction [33] was employed using an ultrasonic bath (USC1200TH, Prolabo, Sion, Switzerland), consisting of a 300 × 240 × 200 mm (inside dimension) tank with an electric power of 400 W equal to an acoustic power of 1 W/cm^2^ and a maximum heating power of 400 W. A frequency controller allowed for the selection of the US frequency of the device, also equipped with a temperature regulator and an automatic digital timer. Using a previously optimized extraction procedure, each sample (50 mg) was suspended in 10 mL 65% (*v/v*) aqueous ethanol and deposited in 50 mL quartz tubes with a vapor condenser and extracted over 40 min at an ultrasound frequency of 30 kHz. Following extraction, each extract was centrifuged for 15 min at 5000× *g* (Heraeus Biofuge Stratos, Thermo Scientific, Illkirch, France), and the supernatant was filtered using a syringe filter (0.45 m, Merck Millipore, Molsheim, France) before analysis. Each experiment was done in triplicate.

### 2.4. Determination of Total Phenolic Content (TPC)

The total phenolic content (TPC) was measured using the Folin–Ciocalteu protocol and microplate spectrophotometry, as described previously [25]. Absorbance was measured at 650 nm with a spectrophotometer (BioTek ELX800 Absorbance Microplate Reader, BioTek Instruments, Colmar, France). A standard curve (0–40 µg/mL; R^2^ = 0.998) of gallic acid (Merck, Saint-Quentin Fallavier, France) was used to express the TPC in mg of gallic acid equivalents per g DW (mg GAE/100 g dry weight (DW)).

### 2.5. Determination of Total Flavonoid Content (TFC)

The colorimetric aluminum trichloride (AlCl_3_) method was used to determine TFC [34]. A 200 µL mixture was made in a microplate using 20 µL of extract, 10 µL of potassium acetate 1 M, 10 µL of AlCl_3_ (10% (*w/v*)), and 160 µL of deionized water. A microplate reader (Multiskan GO, Thermo Fischer Scientific, Illkirch, France) was used to measure the absorbance at 415 nm after 30 min of incubation at 25 °C in the dark. TFC was expressed in mg/100 g dry weight (DW) of quercetin equivalent using a five-point calibration line (linearity range from 0 to 40 g/mL quercetin concentration with an R^2^ of 0.998).

### 2.6. Determination of Total Anthocyanin Content (TAC)

The colorimetric method was used to determine TAC [35]. Absorbance was measured at 510 and 700 nm with a spectrophotometer (BioTek ELX800 Absorbance Microplate Reader, BioTek Instruments, Colmar, France). A standard curve (0–100 µg/mL; R^2^ = 0.999) of cyanidin-3-*O*-glucoside (Merck, Saint-Quentin Fallavier, France) was used to express the TAC in mg of cyanidin-3-*O*-glucoside equivalents per g DW (mg CAE/100 g DW).

### 2.7. HPLC Analysis

Following extraction, each extract was centrifuged for 15 min at 5000× *g* (Heraeus Biofuge Stratos, Thermo Scientific, Illkirch, France), and the supernatant was filtered using a syringe filter (0.45 m, Merck Millipore, Molsheim, France) before analysis. HPLC was used to separate and identify the main isoflavonoids using a Varian system (Varian, Les Ulis, France) that included a Prostar 230 pump, Metachem Degasit, Prostar 410 autosampler, and Prostar 335 Photodiode Array Detector (PAD), and was controlled by Galaxie version 1.9.3.2 software (Varian, Les Ulis, France).

The separation was carried out on a Purospher RP-18 column (250 4.0 mm internal diameter; 5 m) (Merck Chemicals, Molsheim, France) at a temperature of 40 °C. The validated separation conditions were as described previously [36]. The mobile phase was a mixture of water and phosphoric acid (1000:1, *v/v*) (solvent A), and water, acetonitrile, and phosphoric acid (200:800:1, *v/v/v*) (solvent B). During the separation run (including 10 re-equilibration), the mobile phase composition varied according to a linear gradient as follows: B 0% (0 min) to 20% (5 min) to 100% (50 min), followed by 0% (60 min). Between each injection, a 10-min re-equilibration time was applied. The detection of compounds was set at 260 nm (corresponding to the λmax of the main compounds). Quantification was done based on assessment of retention times of commercial standard of daidzein and genistein (Merck, Saint-Quentin Fallavier, France). Since no commercial standard is available for cajanin and cajanol, their contents were quantified using the daidzein standard.

### 2.8. In Vitro Cell Free Antioxidant Assays

The in vitro cell free DPPH (2,2-diphenyl-1-picrylhydrazyl), ABTS (2,2-azinobis(3-ethylbenzthiazoline-6-sulphonic acid) and FRAP (Ferric Reducing Antioxidant Power) assays were used to evaluate the in vitro cell free assays for determining antioxidant activity of the samples using the protocols adapted to the microplate reader (Multiskan GO, Thermo Fischer Scientific, Illkirch, France), as described by Drouet et al. [37] and Tungmunnithum et al. [34].

### 2.9. Yeast Culture Conditions

The yeast strain DBY746 (MAT leu2-3,112 his31 trp1-289a ura3-52 GAI+; ATCC 204660) culture was started with frozen stock plated onto an YPD medium (yeast extract peptone dextrose) (Sigma-Aldrich, Saint-Quentin Fallavier, France). Extracts (at a final concentration of 1 mg/mL) were dissolved in cell culture grade dimethyl sulfoxide (DMSO; Sigma-Aldrich, Saint-Quentin Fallavier, France) and applied at a final DMSO concentration was 0.1% (*v/v*). Control yeast was inoculated with the same DMSO concentration. Resveratrol was used as positive control (at a final concentration of 10 µM). The impact on yeast survival was asserted as previously described [38].

### 2.10. Cellular Antioxidant Assay

Yeast cells were first treated under the same conditions as mentioned above. Yeast cells were irradiated with a UV dose of 106.5 J/m2 UV-C (254 nm) under a Vilber VL-6.C filtered lamp (Thermo Fisher Scientific, Villebon-sur-Yvette, France), and incubated at 28 °C with orbital shaking at 120 rpm in the dark in complete 2.0% (*w/v*) glucose YPD medium (Sigma Aldrich, Saint-Quentin Fallavier, France), as previously described [38]. The same conditions were used to grow non-irradiated cells. Hour 0 of the oxidative stress experiment was considered irradiation.

The dihydrorhodamine-123 (DHR-123) fluorescent dye (Sigma-Aldrich, Saint-Quentin Fallavier, France) was used to assess the quantity of reactive oxygen and nitrogen species. Approximately 10^8^ yeast cells were washed twice in PBS, resuspended in PBS containing 0.4 M DHR-123, and incubated for 10 min in the dark at 28 °C in the presence of extract, RES or DMSO (control cells). The fluorescence signal (ex = 505 nm, em = 535 nm) was measured using the VersaFluor Fluorimeter after two washes with PBS (Biorad, Marnes-la-Coquette, France).

### 2.11. Statistical Analysis

Statistical analyses were performed with XLSTAT 2019 suite (Addinsoft, Paris, France). Data composed of at least three independent replicates were presented using the means and standard deviations. Student’s *t*-test was carried out for statistical comparative analysis. Significant thresholds at *p* < 0.05, 0.01 and 0.001 were represented by *, ** and ***, respectively. Different letters were used to indicate significant thresholds at *p* < 0.05.

## 3. Results and Discussion

### 3.1. Plant Population and Taxonomic Description

After the intense searching for the leaving plant materials in the fields, the 50 Fabaceae populations of the ten species were collected from the different localities covering the entirety of the floristic regions in Thailand for this study, as can be seen in Table 1 and Figure S1.

**Figure 1:**
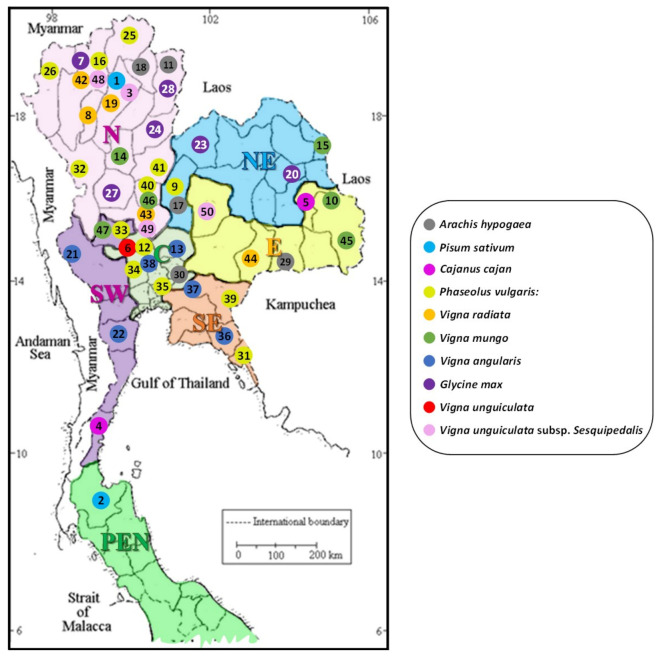
The distribution map of the collected 50 populations of edible seed plants (Fabaceae) throughout Thailand.

The distribution map of the collected 50 populations of edible seed plants (Fabaceae) throughout Thailand is provided in Figure 1. According to the distribution of these 50 populations of the edible seed species of Fabaceae, the most abundant floristic region of these plant group in Thailand is the northern floristic region, in which the 22 populations were found, following by the central and eastern floristic regions, in which the 7 and 6 populations were found, respectively. The less abundant floristic region of these edible seed Fabaceae species belongs to the peninsula floristic region. Furthermore, *Phaseolus vulgaris* is the most abundant species, consisting of 13 populations mainly distributed in the northern (six populations), central (three populations) and south-eastern floristic regions (two populations) in the country, respectively.

The scientific name and the taxonomic description of the collected 50 populations of the edible seed species belonging to the family Fabaceae used in this study are provided below.

**(I)**  *Pisum sativum* L.

Climbing herb, annual, **Stem** glabrous, 0.5–2.0 m tall. **Leaves** stipules, paripinnately compound, leaflets dentate or entire, margin toothed, rachis ending in a branched tendril. **Inflorescence** raceme, 1–3-flowered, papilionaceous form. **Corolla**, usually white and/or purple, vexillum lilac or reddish purple, ovary glabrous, style flat. Fruit legume, seeds 2–10.

**Specimens examined:** Population No. 1 and 2

**(II)**  *Vigna unguiculata* (L.) Walp.

Annual or perennial herb, **Stem** glabrous, erect, or twining, 1.0–3.0 m tall. **Leaves** stipules, paripinnately compound, puberulent or glabrous, leaflets ovate or ovate-rhomboid, base acute to rounded, apex acute. **Inflorescence** raceme, axillary, 2–6 flowered, papilionaceous form. **Corolla**, usually yellowish white or violet, ovary glabrous, style flat. Fruit legume, seeds more than 10, oblong or reniform.

**Specimens examined:** *V. unguiculata*: Population No. 6; *V. unguiculata* subsp. *sesquipedalis*: Population No. 3, 48, 49, and 50

**(III)**  *Cajanus cajan* (L.) Millsp.

Annual shrub, **Stem** erect, glabrous, 1.5–3.0 m tall. **Leaves** stipules, pinnately compound, leaflets ovate-lanceolate, abaxial surface densely pubescent, adaxial surface pubescent, apex acute or acuminate, petiole pubescent. **Inflorescence** raceme, 3–9 flowered, papilionaceous form. **Corolla**, yellow, standard suborbicular, wings obovate, keel apex obtuse, ovary hairy, style long, linear, glabrous, stigma capitate. Fruit legume, seeds 3–7.

**Specimens examined:** Population No. 4 and 5

**(IV)**  *Glycine max* (L.) Merr.

Annual shrub, **Stem** robust, erect, brown, densely hirsute, 0.5–1.0 m tall. **Leaves** stipules, pinnately compound, 3-foliolate, leaflets ovate, broadly ovate, elliptic, or elliptic-lanceolate, base broadly cuneate or rounded, apex acuminate, petiolules hirsute. **Inflorescence** raceme, 3–15 flowered, papilionaceous form. **Corolla**, white, purple or light purple, standard obovate-suborbicular, wings crenate, keel obliquely obovate, ovary hairy, style long, linear, glabrous, stigma capitate. Fruit legume, oblong, densely silky hairy, seeds 2–5.

**Specimens examined:** Population No. 7, 20, 23, 24, 27, and 28

**(V)**  *Vigna radiata* (L.) R.Wilczek

Annual herbs, **Stem** erect, twining, or creeping, hispid with brown spreading hairs, 0.2–0.8 m tall. **Leaves** stipules, pinnately compound, 3-foliolate, leaflets ovate, broadly ovate, base rounded or broadly cuneate, apex acute or acuminate. **Inflorescence** raceme, 25–35 flowered, papilionaceous form. **Corolla**, yellow, yellow-green, standard suboblate, wings ovate, keel falcate, ovary hairy, style long, linear, glabrous, stigma capitate. Fruit legumes, linear-terete, shortly hispid with brown hairs, seeds 8–15.

**Specimens examined**: Population No. 8, 19, 42, 43, and 44

**(VI)**  *Phaseolus vulgaris* L.

Annual herbs, **Stem** suberect, erect or twining, pubescent, 3.5–4.5 m tall. **Leaves** stipules, pinnately compound, 3-foliolate, leaflets broadly ovate or obovate-rhombic, base rounded or broadly cuneate, margin entire, apex acuminate. **Inflorescence** raceme, 15–30 flowered at the top of rachis, papilionaceous form. **Corolla**, white, violet, yellow or red, standard suboblate, wings obovate, keel spirally twisted at apex, ovary pubescent. Fruit legumes, linear or linear-oblong, glabrous, seeds 4–10.

**Specimens examined**: *P. vulgaris* cv. red kidney bean: Population No. 9, 39, 40 and 41 *P. vulgaris* cv. red kidney bean: Population No. 12, 16, 25, 26, 31, 32, 33, 34, and 35

**(VII)**  *Vigna mungo* (L.) Hepper

Annual herbs, **Stem** erect or creeping, hispid with spreading hairs, 0.5–1.0 m tall. **Leaves** stipules, pinnately compound, 3-foliolate, leaflets ovate or broadly ovate, base rounded, apex acute. **Inflorescence** raceme, 20–35 flowered, papilionaceous form. Corolla, yellow, standard suboblate, wings ovate, keel falcate, ovary hairy. Fruit legumes with long hairs, seeds distinctly raised rim-aril around the hilum, 7–15.

**Specimens examined**: Population No. 9, 12, 16, 25, 26, 31, 32, 33, 34, 35, 39, 40, and 41

**(VIII)**  *Vigna angularis* (Willd.) Ohwi & H.Ohashi

Annual herbs, **Stem** erect or twining, angular, 0.3–1.0 m tall. **Leaves** stipules, pinnately compound, 3-foliolate, leaflets rhomboid-ovate, ovate, or broadly ovate, base rounded, apex broadly triangular. **Inflorescence** raceme, 5–8 flowered, papilionaceous form. **Corolla**, yellow, standard oblate or subreniform, wings ovate, broader than keel, keel apex incurved, base clawed, ovary hairy. Fruit legumes with long hairs, seeds distinctly raised rim-aril around the hilum, 7–13.

**Specimens examined**: Population No. 13, 21, 22, 36, 37, and 38

**(IX)**  *Arachis hypogaea* L.

Annual herbs, **Stem** erect or procum bent, yellowish pubescent, glabrescent, 0.3–1.2 m tall. **Leaves** stipules, pinnately compound, 4-foliolate, covered with long flexuous trichomes, leaflets ovate-oblong, obovate, base adnate, apex broadly triangular. **Inflorescence** raceme, 3–5 flowered, papilionaceous form. **Corolla**, yellow, golden yellow, standard spreading, apex emarginate; wings inflexed, apex acuminate, keel acuminate, ovary oblong, style longer than calyx; stigma small, sparsely pubescent. Fruit legumes geocarpic, oblong, seeds oblong, 3–5.

**Specimens examined**: Population No. 11, 17, 18, 29, and 30

### 3.2. Phytochemical Profiles

The total phenolic, flavonoid, and anthocyanin contents (TPC, TFC, and TAC) in the beans of the 50 Thai Fabaceae (i.e., nine species, two subspecies, and four cultivars) populations growing in different areas all differed by more than an order of magnitude, illustrating the high heterogeneity occurring in the phenolics/polyphenols accumulations observed for this plant family (Table 2).

**Figure 2:**
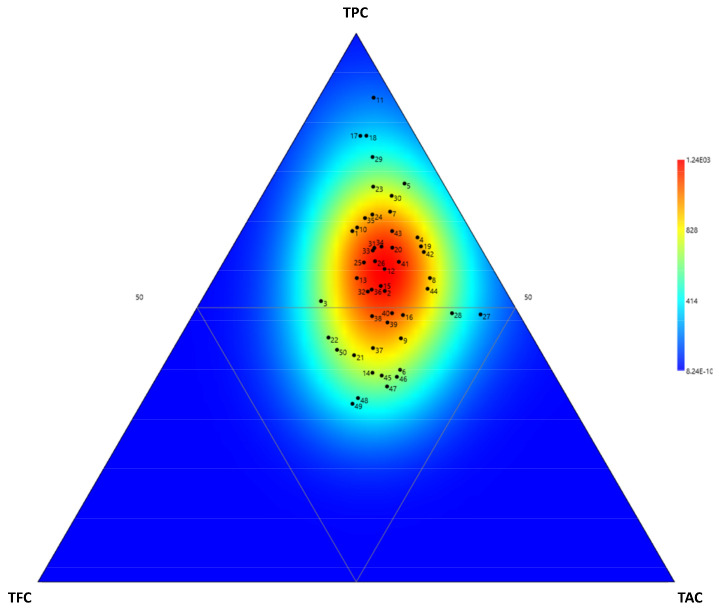
Ternary plot showing the relative TPC, TFC, and TAC within the 50 Fabaceae populations from various floristic regions from Thailand.

The TPC ranged from 56.2 mg/100 g DW (population #2) to 717.3 mg/100 g DW (population #48) gallic acid equivalent. The TFC ranged from 28.0 mg/100 g DW (population #11) to 1536.1 mg/100 g DW (population #49) quercetin equivalent. The TAC ranged from 1.7 g/100 g DW (population #2) to 41.6 g/100 g DW (population #48) cyanidin-3-O-glucoside equivalent. This variation was shown in a ternary plot, which demonstrated the relevance of TPC as a major contributor to phytochemical variation, as evidenced by the heatmap distribution, which was markedly shifted in the top triangle (high TPC) (Figure 2).

In general, the observed ranges of variation for the various species are consistent with published data [11,15,16,23,34].

Within the same species, the variability is also significant, as illustrated for the two *Pisum sativum* cultivars (flat (population #2) vs. round pod (population #1) cv.), which showed great variations for the different types of phytochemicals—with TPC, TFC, and TAC ranging from 56.2 mg/100 g DW to 558.2 mg/100 g DW gallic acid equivalent, 43.1 mg/100 g DW to 348.3 mg/100 g DW quercetin equivalent, and 1.7 mg/100 g DW to 8.8 mg/100 g DW cyanidin equivalent, respectively (Table 2). These ranges of variations are in accordance with data reported by other authors [39,40,41].

*Vigna unguiculata* subsp. *sesquipedalis* populations showed low variability in TPC with contents ranging from 628.3 mg/100 g DW to 717.3 mg/100 g DW gallic acid equivalent, whereas high variability was observed in TFC and TAC ranging from 786.9 mg/100 g DW to 1536.1 mg/100 g DW quercetin equivalent, and 13.4 mg/100 g DW to 41.6 mg/100 g DW cyanidin equivalent (Table 2). We observed that the phenolic compounds in the *Vigna unguiculata* subsp. *sesquipedalis* populations (#2 and #48–50) were higher than in the *Vigna unguiculata* population (#6). Although little is known about the *sesquipedalis* subspecies in the literature, this variability is in reasonable agreement with previous results on *Vigna unguiculata* genotypes from Burkina Faso [42]. In this regard, the present results are noteworthy since they expand the knowledge concerning this subspecies by providing new quantitative information.

The red cultivar population #16 of *Phaseolus vulgaris* had the greatest TAC, but surprisingly the cream cultivars (populations #9, #39–41) were also relatively high in anthocyanins as compared to the other red cultivars (populations #25–26 and #31–35) (Table 2). Similarly, Aquino-Bolaos et al. [43] reported that cream-pink cultivars had the highest anthocyanin content. According to Rodriguez Madrera et al. [23], seed coat colors are the result of multiple potential phytochemical combinations, therefore color categorization does not guarantee that two beans with same-colored coats have similar phenolic compound composition, including anthocyanins.

Phenolic acids, (iso)flavonoids, and anthocyanins are the main phenolic compounds present in legume beans [16,17]. However, isoflavones accumulated in relative high amount in legumes and their derived products; in particular genistein and daidzein are reported as the most physiologically active isoflavones suitable for human health promotion [18,19,20,21]. Indeed, isoflavones are phenolic compounds with a chemical structure similar to estradiol, and can mimic or inhibit the action or metabolism of this essential human hormone [17]. To provide a thorough view of the variations in these two important isoflavones in the beans of the present 50 Fabaceae populations from Thailand, HPLC quantification was performed (Table 2). The variations were huge (1.8 (*Vigna angularis*) to 26,029.9 (*Glycine max*) µg/100 g DW for daidzein; 0.1 (*Pisum sativum*) to 82,514.7 (*Glycine max*) µg/100 g DW for genistein), with *Glycine max* being by far the richest source of both daidzein and genistein. These results are consistent with those found in the literature [17,44], but expand the current knowledge concerning the Thai populations, as well as for some species such *Vigna unguiculata* subsp. *sesquipedalis*.

**Figure 3:**
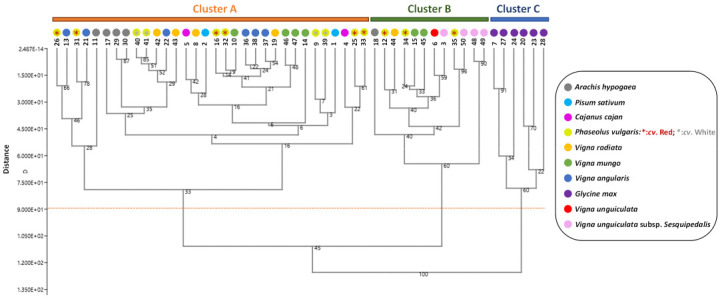
Hierarchical clustering analysis dendrogram according to the phytochemical composition of the extracts of 50 Fabaceae populations from various floristic regions from Thailand. The percentages of replicate trees in which associated samples cluster together in the bootstrap test (percentage of 5000 replicates) are shown next to the branches.

Cluster analysis was used to discern possible groups among the various populations (Figure 3).

Based on their phytochemical profiles, the hierarchical cluster analysis (HCA) divides the 50 populations into three major clusters (A, B and C, Figure 3). Despite the fact that the 50 populations were composed of 10 identifiable species, the cluster analysis revealed no evident pattern showing a prominence for this genetic background factor. Some were more homogeneous species, such as cluster C composed of *Glycine max* populations (the last population of this species was grouped in cluster A), while other species, such as *Vigna mungo* or *Phaseolus vulgaris*, showed more heterogeneity and were found throughout the three clusters. For instance, the heterogeneinty of species has been already reported [23,43]. It is worth noting that, in addition to genetics, environmental factors such as climatic and geographic (including soil conditions) factors have been shown to have a significant influence on the accumulation of phenolic compounds [26,37]. This has already been observed in populations of *Medicago minima* from the Fabaceae family [45]. As a consequence, given the large geographic distribution throughout the different floristic regions of Thailand of the present 50 Fabaceae populations, environmental factors might explain at least some part of the variability in phytochemical profiles observed within the same species.

Our results reveal a comprehensive picture of the various phenolic compounds that may have a health-promoting effect on humans within the beans of 50 edible Fabaceae populations from Thailand. The next step was to investigate towards how their antioxidant potential varied.

### 3.3. Antioxidant Potential

Table 3 shows the results of the evaluation of the antioxidant activity estimated using both in vitro cell-free assays (DPPH, ABTS and FRAP, expressed in µmol of Trolox equivalent antioxidant capacity) and cellular antioxidant assay (CAA, expressed in inhibition percentage of reactive oxygen and nitrogen species (ROS/RNS)) in yeast cells subjected to UV-induced oxidative stress.

The DPPH free radical scavenging activity of the extracts ranged from 27.0 (*A. hypogaea*, population #17) to 84.1 (*V. angularis*, population #22) µmol TE/g DW. The ABTS radical scavenging activity ranged from 39.4 (*P. sativum*, cv. Flat pod, population #2) to 114.5 (*V. angularis*, population #22) µmol TE/g DW. The FRAP reducing power ranged from 45.0 (*C. cajan*, population #5) to 331.2 (*V. unguiculata* subsp. *sesquipedalis*, population #50) µmol TE/g DW.

The antioxidant activity of plant extracts cannot be assessed using a single approach due to the complex nature of phytochemicals, and, in particular, since the determination of antioxidant activity is strongly dependent on the reaction mechanism involved [46,47]. Several chemical or biological tests are necessary to measure antioxidant activity and establish the antioxidant mechanism of action of a plant extract [46,47]. The chemical reactions on which the in vitro cell free antioxidant assays are based may be categorized into 3 types: the ABTS assay is based on a hydrogen atom transfer reaction (HAT), the FRAP assay is based on an electron transfer reaction (ET), and the DPPH assay is a combination of both mechanism [37,46,47,48].

**Figure 4:**
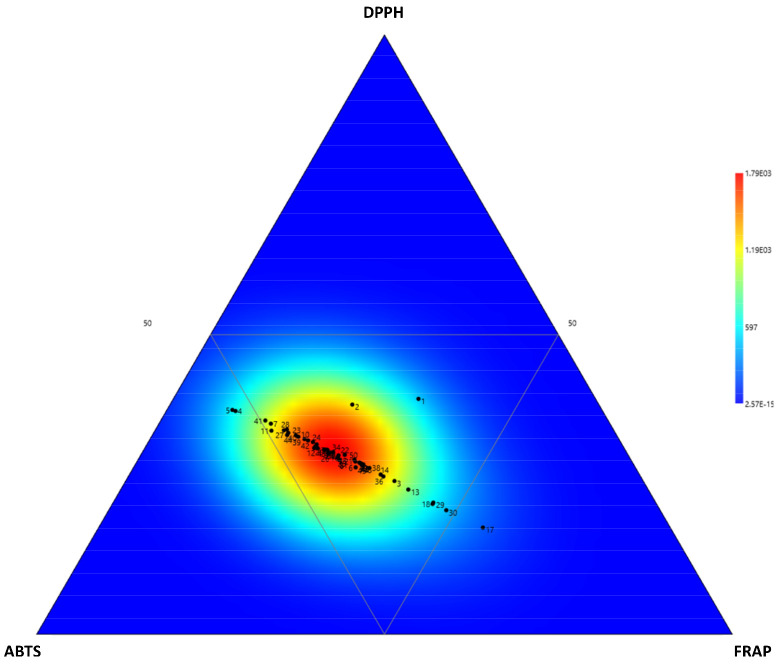
Ternary plot showing the contribution of the relative in vitro assay to the antioxidant activity of the 50 Fabaceae populations from various floristic regions from Thailand.

Here, the antioxidant capacity of the FRAP assay was higher than that of the ABTS and DPPH assays. These results might hint to an antioxidant capacity mediated by an ET-type mechanism rather than a HAT-type mechanism (Figure 4).

It also appears that the in vitro cell-free antioxidant capacity varies significantly within the same species and/or cultivar (Figure 4). These observations might be explained by the huge variations observed in individual composition in antioxidant phytochemicals (Table 2). This has previously been reported within for a single Fabaceae species, such as in *M. minima* [45], *P. vulgaris* [23,43], *P. sativum* [39,41], or *V. unguiculata* [42]. Our study here is performed at the population level of diverse Fabaceae species, thus contributing to a better knowledge about the variation of antioxidant capacity in this plant family.

Although in vitro assays are useful for predicting chemical mechanisms, they may not always correlate with the in vivo antioxidant capacity of an extract. Thus, the validity of these in vitro cell-free antioxidant assays must be limited to the chemical reactivity interpretation and in vivo validation is required. Therefore, here, CAA was also considered (Table 3). The CAA widely varied and ranged from 59.7% (*C. cajan*, population #5) to 87.9% (*V. unguiculata* subsp. *sesquipedalis*, population #50) of ROS/RNS inhibition.

Interestingly in vivo CAA results showed good correlation with the in vitro cell-free FRAP (HAT) antioxidant assay.

Plant (poly)phenols are powerful natural antioxidants found in food that have been proven to protect cells against the damaging effects of excessive ROS and RNS production [49,50].

Here, yeast cells have been used for CAA. Yeast cells have been widely used as a model for assessing antioxidant capacity of various extracts or compounds [38,51,52]. It is a reliable eukaryotic model with well-known mechanisms involved in defense and/or adaptation to oxidative stress that can be easily expanded to human due to molecular mechanism well conserved within eukaryotic cells [51,52]. The production of ROS and RNS increases with age, stress, or pollution as a direct result of redox cellular imbalances, and have been linked with aging processes and possibly contribute to the development of a variety of degenerative diseases [50,53,54]. Thus, the present results supported the possible protective effect described for Fabaceae phenolics against chronic degenerative diseases [55], and constitute the most complete database at population level of different species/cultivar from Thailand. These results may be valuable in selecting the starting material for breeding, as well as producing antioxidant extracts suitable for applications as nutraceuticals or cosmeceuticals.

### 3.4. Correlation Analysis

**Figure 5:**
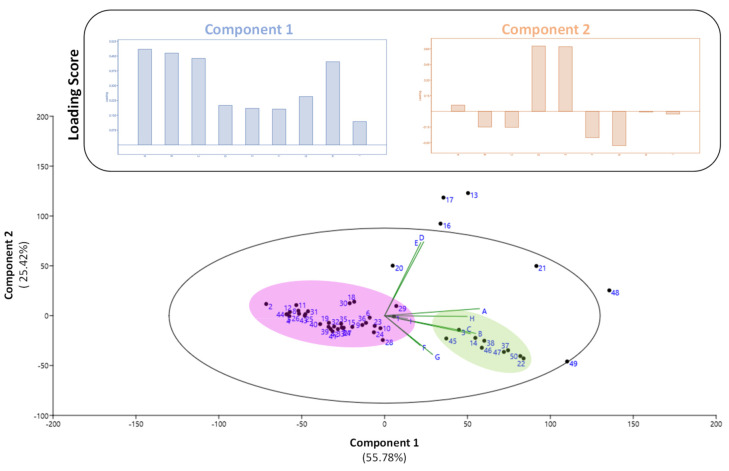
Principal component analysis (PCA) linking the phytochemical profile and (in vitro and cellular) antioxidant capacity of the extracts from 50 Fabaceae populations from Thailand. Variance of component 1 = 55.78% and component = 25.42%. Each number in blue represents the different Fabaceae populations (Figure 1). Letters represent the different phytochemicals and antioxidant assays: A = TPC; B = TFC; C = TAC; D = daidzein content; E = genistein content; F = DPPH assay; G= ABTS assay; H = FRAP assay; I = CAA.

A principal component analysis (PCA) was used with the variables related with phytochemical composition and antioxidant activity to determine different groups among the extracts from in the 50 Fabaceae populations (Figure 5).

The resulting biplot representation accounts for 81.20% (component 1 + component 2) of the original variability of the data (Figure 5). Discrimination occurs mostly in the first dimension (component 1 axis), which accounts for 55.78% of the initial variability, with the phytochemicals TPC, TFC, TAC, and FRAP antioxidant test as the main contributors (see the loading scores for component 1). The two other phytochemicals investigated (daidzein and genistein) appeared to contribute to the second dimension (component 2 axis), although only to a small extent (25.42% of the original variability). As a consequence, PCA revealed that two major clusters were significantly different from one another in terms of phytochemical profile and antioxidant activity. Interestingly, the green cluster, which is relatively rich in TPC, TFC, and TAC and has strong FRAP antioxidant activity, is solely made up of extracts from *Vigna* species (i.e., four *V. mungo* populations (#14, #45, #46 and #47), three *V. angularis* populations (#22, #37 and #38), one *V. unguiculata* population (#3) and one *V. unguiculata* subsp. *sesquipedalis* population (#50)), indicating that this clustering might be directly connected to genetic variability of Fabaceae genotypes from Thailand.

**Figure 6:**
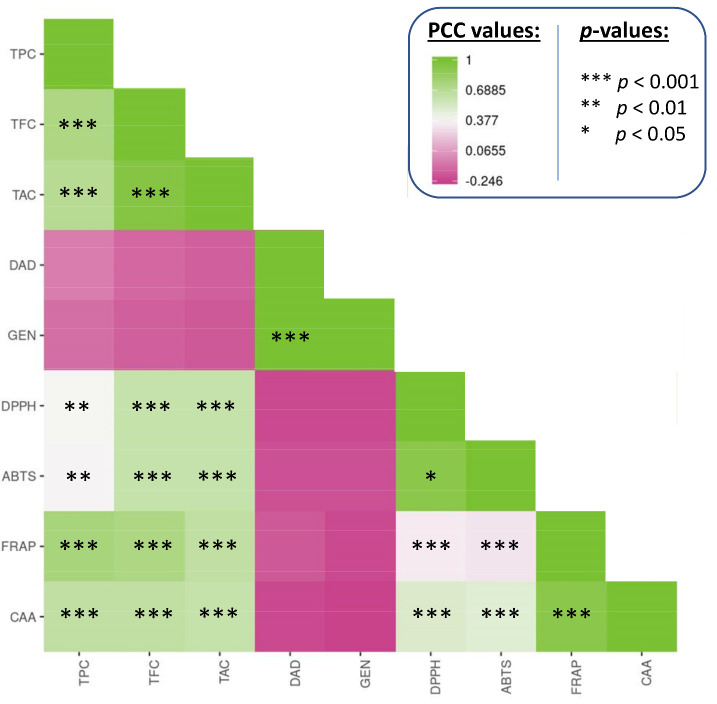
Correlogram analysis (Pearson coefficient correlation) between phytochemical profiles and antioxidant activities of extracts. *** significant *p* < 0.001; ** significant *p* < 0.01; * significant *p* < 0.05; PCC values are indicated in Table S1.

Finally, Pearson correlation coefficients (PCC) were calculated, and a matrix was created to evaluate the association between antioxidant activity and phytochemical profile within the extracts from the 50 populations (Figure 6, Table S1).

This analysis clearly confirmed the strength of the relationship between several variables such as TPC, TFC, and TAC and the different antioxidant assays, in particular FRAP assay. The observed predominance of FRAP and CAA assays could be explained by the fact that all of these phytochemicals (i.e., TPC, TFC, and TAC) strongly contribute to these two antioxidant activities. The absence of correlation between the antioxidant assays and the isoflavones daidzein and genistein concentrations might be surprising. This can be explained in part by the fact that the antioxidant capacity of an extract is the product of complex phytochemical combinations [47]. Furthermore, here, only *G. max* extracts were found to be rich in both daidzein and genistein, which may have skewed the correlation study for these two isoflavones. This was confirmed by looking at the intraspecific PCC calculated within a same species/subspecies (Table S2). Finally, daidzein and genistein are recognized phytoestrogens, but the human metabolites formed as a result of their consumption are more effective antioxidants than these plant-derived parent chemicals [56].

## 4. Conclusions

The 50 populations of Fabaceae plant family throughout all the floristic regions in Thailand showed the great variation in their phytochemical profiles, as well as antioxidant capacity. However, this cellular antioxidant result showed good correlation with the in vitro cell-free FRAP antioxidant assay to point out that antioxidant capacity of these Fabaceae populations mediated by an electron transfer mechanism. It is clearly seen that the Fabaceae edible species are varied in their phytochemicals and antioxidant potential both at in vitro and *in cellulo* levels. Thus, the findings of this study can be applied to the nutraceutical and phytopharmaceutical sectors for their consideration of the best population to use as the potential raw plant material for product development. Furthermore, the future studies on other biological activities, as well as the toxicity test, should be investigated to discover the potential of Fabaceae plants for nutraceutical and novel food applications for product development.

## Figures and Tables

**Table 1:** The 50 populations of edible seed plants (Fabaceae) throughout Thailand.

Population No.	Collected Sites	Floristic Regions	Plant Species	Color of Testa
1	Lampang	N	*Pisum sativum* L. cv. round pod	Light green
2	Surat Thani	PEN	*Pisum sativum* L. cv. flat pod	Light green
3	Lampang	N	*Vigna unguiculata* subsp. *sesquipedalis* (L.) Verdc.	Dark red
4	Chumphon	SW	*Cajanus cajan* (L.) Millsp.	Cream with brown spots
5	Yasothon	E	*Cajanus cajan* (L.) Millsp.	Cream with brown spots
6	Chainat	C	*Vigna unguiculata* subsp. *unguiculata* (L.) Walp.	Dark red
7	Chiang Mai	N	*Glycine max* (L.) Merr.	Light yellow
8	Lamphun	N	*Vigna radiata* (L.) R.Wilczek	Green
9	Phetchabun	NE	*Phaseolus vulgaris* L. cv. white kidney beans	White
10	Ubon Ratchathani	E	*Vigna mungo* (L.) Hepper	Black
11	Nan	N	*Arachis hypogaea* L.	Light brown
12	Chainat	C	*Phaseolus vulgaris* L. cv. red kidney bean	Dark red
13	Lop Buri	C	*Vigna angularis* (Willd.) Ohwi & H.Ohashi	Dark red
14	Sukhothai	N	*Vigna mungo* (L.) Hepper	Black
15	Nakhon Phanom	NE	*Vigna mungo* (L.) Hepper	Black
16	Chiang Mai	N	*Phaseolus vulgaris* L. cv. red kidney bean	Dark red
17	Phetchabun	NE	*Arachis hypogaea* L.	Light brown
18	Phayao	N	*Arachis hypogaea* L.	Light brown
19	Lampang	N	*Vigna radiata* (L.) R.Wilczek	Green
20	Kalasin	NE	*Glycine max* (L.) Merr.	Light yellow
21	Kanchanaburi	SW	*Vigna angularis* (Willd.) Ohwi & H.Ohashi	Dark red
22	Phetchaburi	SW	*Vigna angularis* (Willd.) Ohwi & H.Ohashi	Dark red
23	Loei	NE	*Glycine max* (L.) Merr.	Light yellow
24	Uttaradit	N	*Glycine max* (L.) Merr.	Light yellow
25	Chiang Rai	N	*Phaseolus vulgaris* L. cv. red kidney bean	Dark red
26	Mae Hong Son	N	*Phaseolus vulgaris* L. cv. red kidney bean	Dark red
27	Kamphaeng Phet	N	*Glycine max* (L.) Merr.	Light yellow
28	Nan	N	*Glycine max* (L.) Merr.	Light yellow
29	Surin	E	*Arachis hypogaea* L.	Light brown
30	Nakhon Nayok	C	*Arachis hypogaea* L.	Light brown
31	Trat	SE	*Phaseolus vulgaris* L. cv. red kidney bean	Dark red
32	Tak	N	*Phaseolus vulgaris* L. cv. red kidney bean	Dark red
33	Uthai Thani	SW	*Phaseolus vulgaris* L. cv. red kidney bean	Dark red
34	Suphan Buri	C	*Phaseolus vulgaris* L. cv. red kidney bean	Dark red
35	Pathum Thani	C	*Phaseolus vulgaris* L. cv. red kidney bean	Dark red
36	Chanthaburi	SE	*Vigna angularis* (Willd.) Ohwi & H.Ohashi	Dark red
37	Prachin Buri	SE	*Vigna angularis* (Willd.) Ohwi & H.Ohashi	Dark red
38	Ang Thong	C	*Vigna angularis* (Willd.) Ohwi & H.Ohashi	Dark red
39	Prachin Buri	SE	*Phaseolus vulgaris* L. cv. white kidney beans	White
40	Phichit	N	*Phaseolus vulgaris* L. cv. white kidney beans	White
41	Phitsanulok	N	*Phaseolus vulgaris* L. cv. white kidney beans	White
42	Chiang Mai	N	*Vigna radiata* (L.) R.Wilczek	Green
43	Nakhon Sawan	N	*Vigna radiata* (L.) R.Wilczek	Green
44	Buriram	E	*Vigna radiata* (L.) R.Wilczek	Green
45	Ubon Ratchathani	E	*Vigna mungo* (L.) Hepper	Black
46	Phichit	N	*Vigna mungo* (L.) Hepper	Black
47	Uthai Thani	SW	*Vigna mungo* (L.) Hepper	Black
48	Chiang Mai	N	*Vigna unguiculata* subsp. *sesquipedalis* (L.) Verdc.	Dark red
49	Nakhon Sawan	N	*Vigna unguiculata* subsp. *sesquipedalis* (L.) Verdc.	Dark red
50	Chaiyaphum	E	*Vigna unguiculata* subsp. *sesquipedalis* (L.) Verdc.	Dark red

**Table 2:** Phytochemical profiles of 50 Fabaceae populations from various floristic regions from Thailand.

Population Number (Plant Species)		TPC (mg/100 g DW)	TFC (mg/100 g DW)	TAC (mg/100 g DW)	DAD (µg/100 g DW)	GEN (µg/100 g DW)
11	(*Arachis hypogaea*)	368.7 ± 11.9 fg	28.0 ± 0.2 k	2.1 ± 0.1 l	17.3 ± 0.2 d	38.5 ± 5.2 g
17	(*Arachis hypogaea*)	686.8 ± 36.5 ab	157.2 ± 11.9 h	4.9 ± 0.6 k	54.2 ± 0.6 b	80.2 ± 0.9 e
18	(*Arachis hypogaea*)	488.5 ± 23.3 d	99.5 ± 2.4 i	3.8 ± 0.2 k	44.3 ± 0.4 b	76.5 ± 0.7 e
29	(*Arachis hypogaea*)	670.8 ± 6.2 ab	161.3 ± 12.5 gh	7.0 ± 0.5 j	56.7 ± 6.1 b	80.8 ± 0.9 e
30	(*Arachis hypogaea*)	370.9 ± 1.9 fg	104.3 ± 10.4 i	6.2 ± 0.5 j	50.9 ± 4.2 b	79.5 ± 0.5 e
4	(*Cajanus cajan*)	201.4 ± 23.6 ij	61.7 ± 2.3 j	5.3 ± 0.5 jk	13.5 ± 0.3 d	1.2 ± 0.3 k
5	(*Cajanus cajan*)	232.1 ± 20.8 i	41.7 ± 1.2 j	3.9 ± 0.7 k	10.3 ± 0.2 e	0.8 ± 0.3 l
7	(*Glycine max*)	358.3 ± 21.8 fg	124.2 ± 14.6 h	6.6 ± 1.5 j	14,238.5 ± 248.6 a	48,564.2 ± 50.6 b
20	(*Glycine max*)	483.4 ± 13.3 d	235.6 ± 22.0 g	11.6 ± 1.0 i	26,029.9 ± 233.3 a	82,514.7 ± 267.3 a
23	(*Glycine max*)	549.9 ± 24.2 c	184.7 11.3 gh	7.4 ± 0.4 j	21,524.7 ± 268.4 a	67,289.8 ± 196.1 a
24	(*Glycine max*)	401.0 ± 22.3 f	179.2 ± 15.2 gh	6.6 ± 0.3 j	20,356.7 ± 266.6 a	68,567.8 ± 163.0 a
27	(*Glycine max*)	277.0 ± 11.1 hi	74.0 ± 3.2 ij	14.9 ± 0.3 h	9900.2 ± 232.1 a	39,562.6 ± 139.8 b
28	(*Glycine max*)	359.1 ± 21.8 fg	164.1 ± 11.0 gh	17.3 ± 0.4 g	20,412.3 ± 261.7 a	40,785.0 ± 150.7 b
12	(*Phaseolus vulgaris* (red kidney beans)	115.7 ± 8.2 l	74.0 ± 1.1 i	3.1 ± 0.3 l	3.2 ± 0.3 h	30.2 ± 1.8 g
16	(*Phaseolus vulgaris* (red kidney beans))	440.2 ± 12.7 c	355.2 ± 24.4 ef	17.3 ± 2.8 g	7.3 ± 1.2 f	129.2 ± 6.2 d
25	(*Phaseolus vulgaris* (red kidney beans))	137.5 ± 10.3 kl	99.5 ± 4.5 i	3.0 ± 0.5 l	3.7 ± 0.4 g	33.1 ± 0.2 g
26	(*Phaseolus vulgaris* (red kidney beans))	125.0 ± 10.2 kl	81.6 ± 2.3 i	2.9 ± 0.6 l	3.6 ± 0.3 g	34.8 ± 0.2 g
31	(*Phaseolus vulgaris* (red kidney beans))	192.6 ± 10.5 i	113.2 ± 9.5 i	4.1 ± 0.9 k	3.8 ± 0.4 g	41.2 ± 0.3 f
32	(*Phaseolus vulgaris* (red kidney beans))	164.0 ± 12.4 k	144.1 ± 12.8 gh	4.6 ± 1.1 k	3.6 ± 0.6 g	39.5 0.2 f
33	(*Phaseolus vulgaris* (red kidney beans))	193.8 ± 11.6 i	118.0 ± 11.5 i	4.2 ± 0.2 k	3.4 ± 0.5 g	39.4 ± 0.3 f
34	(*Phaseolus vulgaris* (red kidney beans))	200.2 ± 10.6 i	108.4 ± 14.5 i	4.4 ± 0.3 k	3.2 ± 0.4 g	37.5 ± 0.3 f
35	(*Phaseolus vulgaris* (red kidney beans))	240.7 ± 22.8 i	120.1 ± 16.7 i	3.8 ± 0.6 kl	3.9 ± 0.5 g	44.3 ± 3.5 f
9	(*Phaseolus vulgaris* (white kidney beans))	305.1 ± 21.3 h	305.7 ± 23.3 f	13.9 ± 2.9 h	7.1 ± 1.1 f	118.6 ± 4.4 j
39	(*Phaseolus vulgaris* (white kidney beans))	173.3 ± 10.5 k	168.2 ± 18.1 gh	6.6 ± 1.0 j	4.5 ± 0.6 g	56.3 ± 2.6 f
40	(*Phaseolus vulgaris* (white kidney beans))	180.2 ± 13.4 k	156.5 ± 17.9 h	6.6 ± 1.8 j	4.3 ± 0.6 g	50.2 ± 0.3 f
41	(*Phaseolus vulgaris* (white kidney beans))	244.8 ± 17.9 i	127.0 ± 18.6 h	6.7 ± 1.8 j	4.0 ± 0.5 g	45.7 ± 0.4 f
2	(*Pisum sativum* (flat pod))	56.2 ± 2.1 m	43.1 ± 1.1 j	1.7 ± 0.4 l	13.4 ± 0.2 d	0.2 ± 0.1 m
1	(*Pisum sativum* (round pod))	558.2 ± 44.3 c	348.3 ± 24.3 ef	8.8 ± 2.0 j	28.6 ± 1.2 c	0.3 ± 0.8 m
13	(*Vigna angularis*)	499.2 ± 3.5 d	428.7 ± 16.4 e	11.7 ± 0.9 i	2.3 ± 1.5 i	0.7 ± 0.3 l
21	(*Vigna angularis*)	643.2 ± 25.7 b	988.9 ± 32.9 b	26.2 ± 2.3 d	6.4 ± 0.3 f	2.3 ± 0.9 j
22	(*Vigna angularis*)	697.7 ± 36.7 ab	1076.2 ± 38.9 b	21.2 ± 0.4 e	6.5 ± 0.6 f	2.5 ± 1.0 j
36	(*Vigna angularis*)	254.7 ± 19.9 i	214.3 ± 21.7 gh	7.1 ± 1.1 j	1.8 ± 0.8 i	0.5 ± 0.4 l
37	(*Vigna angularis*)	650.8 ± 35.8 b	851.5 ± 24.5 c	27.7 ± 3.7 d	6.0 ± 2.9 f	2.5 ± 0.9 j
38	(*Vigna angularis*)	668.5 ± 26.1 ab	687.9 ± 16.1 d	22.6 ± 3.4 e	5.2 ± 2.3 f	2.1 ± 0.9 j
10	(*Vigna mungo*)	501.8 ± 13.5 d	291.9 ± 12.0 f	8.0 ± 1.2 j	14.6 ± 1.0 d	18.0 ± 0.7 h
14	(*Vigna mungo*)	547.2 ± 14.1 c	872.1 ± 25.7 c	27.9 ± 1.2 d	31.2 ± 2.9 c	53.2 ± 0.8 f
15	(*Vigna mungo*)	263.5 ± 1.0 i	200.5 ± 11.5 g	7.6 ± 0.4 j	14.3 ± 0.7 d	17.9 ± 0.4 h
45	(*Vigna mungo*)	445.5 ± 12.8 e	689.2 ± 16.1 d	24.2 ± 6.4 e	25.5 ± 2.3 c	48.1 ± 0.6 f
46	(*Vigna mungo*)	509.0 ± 23.6 cd	726.4 ± 17.9 d	29.8 ± 5.3 c	27.6 ± 2.5 c	52.4 ± 0.7 f
47	(*Vigna mungo*)	545.4 ± 14.1 c	896.2 ± 27.9 c	32.9 ± 5.8 b	29.2 ± 3.0 c	56.3 ± 0.8 f
8	(*Vigna radiata*)	133.9 ± 7.3 kl	55.5 ± 2.5 j	4.8 ± 0.8 k	44.3 ± 0.3 b	234.5 ± 20.7 c
19	(*Vigna radiata*)	199.1 ± 10.6 j	64.4 ± 1.2 ij	5.6 ± 0.3 k	48.8 ± 0.3 b	276.5 ± 2.9 c
42	(*Vigna radiata*)	171.7 ± 4.4 k	56.8 ± 3.2 j	5.1 ± 1.2 k	46.6 ± 2.6 b	245.1 ± 2.6 c
43	(*Vigna radiata*)	160.4 ± 12.3 k	66.5 ± 3.1 j	3.4 ± 0.9 kl	49.1± 3.0 b	298.2 ± 2.4 c
44	(*Vigna radiata*)	107.6 ± 8.2 l	52.0 ± 4.1 j	4.0 ± 0.9 k	41.2 ± 2.5 b	213.7 ± 1.7 c
3	(*Vigna unguiculata* subsp. *Sesquipedalis*)	628.3 ± 35.4 b	786.9 ± 21.0 c	13.4 ± 1.9 h	27.3 ± 2.7 c	8.9 ± 0.9 i
48	(*Vigna unguiculata* subsp. *Sesquipedalis*)	717.3 ± 37.1 a	1510.7 ± 76.1 a	41.6 ± 7.2 a	54.3 ± 5.1 b	16.7 ± 1.0 h
49	(*Vigna unguiculata* subsp. *Sesquipedalis*)	678.1 ± 26.3 ab	1536.1 ± 78.8 a	40.2 ± 8.6 a	53.5 ± 5.1 b	16.5 ± 0.9 h
50	(*Vigna unguiculata* subsp. *Sesquipedalis*)	658.3 ± 25.9 ab	1061.1 ± 37.8 b	23.3 ± 5.8 e	42.4 ± 3.6 b	15.7 ± 0.9 h
6	(*Vigna unguiculata*)	337.6 ± 14.6 gh	444.5 ± 16.8 e	19.0 ± 1.5 f	22.9 ± 1.5 cd	8.1 ± 0.5 i

Different letters indicate significant differences at *p* < 0.05.

**Table 3:** In vitro cell-free and cellular antioxidant activity of ethanolic extracts of 50 Fabaceae populations from various floristic regions from Thailand.

Population Number (Plant Species)		DPPH (µmol TE/g DW)	ABTS (µmol TE/g DW)	FRAP (µmol TE/g DW)	CAA (% ROS/RNS Inhibition)
11	(*Arachis hypogaea*)	37.5 ± 1.5 l	54.3 ± 3.1 i	71.7 ± 3.4 j	61.4 ± 6.0 c
17	(*Arachis hypogaea*)	27.0 ± 1.1 m	40.9 ± 2.2 k	325.4 ± 7.9 a	84.0 ± 6.2 a
18	(*Arachis hypogaea*)	31.3 ± 1.4 m	46.4 ± 2.8 j	257.5 ± 8.2 c	81.2 ± 7.4 a
29	(*Arachis hypogaea*)	36.1 ± 1.1 l	52.6 ± 2.2 i	294.1 ± 4.8 b	83.5 ± 5.2 a
30	(*Arachis hypogaea*)	30.6 ± 1.1 m	45.5 ± 2.2 j	279.1 ± 4.4 b	82.2 ± 5.0 ab
4	(*Cajanus cajan*)	47.2 ± 0.4 i	66.9 ± 0.8 g	49.0 ± 6.9 k	61.3 ± 3.8 c
5	(*Cajanus cajan*)	46.1 ± 0.4 i	65.4 ± 0.8 g	45.0 ± 8.5 k	59.7 ± 4.4 c
7	(*Glycine max*)	61.1 ± 1.4 e	84.7 ± 2.7 e	108.6 ± 8.8 i	75.2 ± 7.5 ab
20	(*Glycine max*)	48.1 ± 1.3 i	68.1 ± 2.6 g	145.5 ± 5.2 fg	75.9 ± 5.9 ab
23	(*Glycine max*)	59.8 ± 1.4 ef	83.2 ± 2.7 e	144.5 ± 5.2 fg	78.1 ± 6.1 ab
24	(*Glycine max*)	66.6 ± 1.2 d	91.9 ± 2.4 a	191.0 ± 4.3 de	81.9 ± 5.3 ab
27	(*Glycine max*)	57.4 ± 1.5 f	80.0 ± 3.0 ef	121.2 ± 4.3 g	75.6 ± 6.2 ab
28	(*Glycine max*)	71.1 ± 1.7 c	97.7 ± 3.3 cd	152.6 ± 5.5 f	80.4 ± 7.2 ab
12	(*Phaseolus vulgaris* (red kidney beans)	41.5 ± 1.0 j	59.5 ± 2.0 h	126.4 ± 4.5 g	72.2 ± 4.7 b
16	(*Phaseolus vulgaris* (red kidney beans))	51.8 ± 1.0 h	72.8 ± 2.1 f	179.6 ± 3.8 e	79.2 ± 4.6 ab
25	(*Phaseolus vulgaris* (red kidney beans))	43.9 ± 1.6 j	62.7 ± 3.3 h	165.7 ± 7.7 ef	76.8 ± 8.0 ab
26	(*Phaseolus vulgaris* (red kidney beans))	43.6 ± 2.1 j	62.2 ± 4.2 h	150.5 ± 8.7 f	75.4 ± 9.7 ab
31	(*Phaseolus vulgaris* (red kidney beans))	40.3 ± 1.4 k	58.0 ± 2.9 h	155.5 ± 3.7 f	75.1 ± 5.8 b
32	(*Phaseolus vulgaris* (red kidney beans))	58.1 ± 1.8 fg	80.9 ± 3.6 ef	194.0 ± 4.5 de	81.1 ± 7.2 ab
33	(*Phaseolus vulgaris* (red kidney beans))	62.3 ± 0.9 e	86.3 ± 1.8 e	186.3 ± 8.1 e	81.2 ± 5.8 ab
34	(*Phaseolus vulgaris* (red kidney beans))	60.5 ± 0.8 e	84.1 ± 1.5 e	212.4 ± 7.7 d	82.3 ± 5.2 ab
35	(*Phaseolus vulgaris* (red kidney beans))	56.0 ± 1.0 g	78.2 ± 2.0 ef	207.4 ± 4.6 d	81.5 ± 4.8 ab
9	(*Phaseolus vulgaris* (white kidney beans))	51.8 ± 1.1 h	72.8 ± 2.3 f	169.4 ± 1.7 e	78.5 ± 4.2 a
39	(*Phaseolus vulgaris* (white kidney beans))	57.9 ± 1.4 fg	80.7 ± 2.8 ef	144.4 ± 5.9 fg	77.7 ± 6.5 b
40	(*Phaseolus vulgaris* (white kidney beans))	54.7 ± 1.3 g	76.5 ± 2.6 f	122.3 ± 7.8 g	75.1 ± 6.9 b
41	(*Phaseolus vulgaris* (white kidney beans))	64.8 ± 1.8 d	89.5 ± 3.7 de	106.0 ± 8.8 i	75.8 ± 8.9 b
2	(*Pisum sativum* (flat pod))	42.6 ± 0.5 j	39.4 ± 0.9 k	113.1 ± 3.6 gh	67.7 ± 4.6 b
1	(*Pisum sativum* (round pod))	69.8 ± 1.9 cd	61.6 ± 3.8 gh	243.3 ± 8.0 c	83.2 ± 8.8 a
13	(*Vigna angularis*)	43.2 ± 1.3 j	61.7 ± 2.6 h	287.2 ± 4.9 b	83.9 ± 5.8 a
21	(*Vigna angularis*)	66.3 ± 0.7 j	91.5 ± 1.4 d	280.5 ± 8.9 b	85.6 ± 5.3 a
22	(*Vigna angularis*)	84.1 ± 0.8 a	114.5 ± 1.6 a	320.0 ± 7.2 a	87.8 ± 5.0 a
36	(*Vigna angularis*)	52.1 ± 1.4 h	73.2 ± 2.9 f	282.0 ± 5.5 b	84.5 ± 6.5 a
37	(*Vigna angularis*)	73.5 ± 1.5 c	100.7 ± 3.1 bc	320.9 ± 5.3 a	87.3 ± 6.8 a
38	(*Vigna angularis*)	66.1 ± 1.5 d	91.3 ± 3.0 de	314.5 ± 6.0 ab	86.6 ± 6.9 a
10	(*Vigna mungo*)	60.7 ± 1.8 e	84.2 ± 1.9 e	160.7 ± 5.0 ef	79.4 ± 4.9 a
14	(*Vigna mungo*)	56.4 ± 1.5 g	78.7 ± 3.0 f	296.7 ± 4.7 b	85.4 ± 6.4 a
15	(*Vigna mungo*)	57.6 ± 0.9 fg	80.3 ± 1.9 f	191.5 ± 2.4 de	80.8 ± 3.8 a
45	(*Vigna mungo*)	60.1 ± 1.6 ef	83.5 ± 3.2 e	271.0 ± 3.3 bc	84.8 ± 6.2 a
46	(*Vigna mungo*)	69.2 ± 1.6 ef	95.2 ± 3.1 d	305.6 ± 5.0 ab	86.6 ± 6.7 a
47	(*Vigna mungo*)	71.2 ± 1.2 c	97.8 ± 2.3 cd	315.1 ± 4.3 ab	87.0 ± 5.2 a
8	(*Vigna radiata*)	39.6 ± 1.2 kl	57.0 ± 2.4 hi	153.3 ± 1.8 f	74.7 ± 4.3 ab
19	(*Vigna radiata*)	54.5 ± 1.4 g	76.3 ± 2.9 f	179.3 ± 5.5 e	79.7 ± 6.5 a
42	(*Vigna radiata*)	62.4 ± 1.6 de	86.5 ± 3.3 e	171.1 ± 8.6 ef	80.3 ± 8.3 ab
43	(*Vigna radiata*)	46.5 ± 1.4 i	66.0 ± 2.9 g	144.4 ± 4.3 fg	75.4 ± 6.1 b
44	(*Vigna radiata*)	44.4 ± 1.4 ij	63.3 ± 2.7 gh	100.6 ± 3.7 i	69.7 ± 5.6 b
3	(*Vigna unguiculata* subsp. *Sesquipedalis*)	54.8 ± 0.5 g	76.7 ± 1.0 f	321.7 ± 6.8 a	86.1 ± 4.0 a
48	(*Vigna unguiculata* subsp. *Sesquipedalis*)	72.7 ± 1.1 c	99.7 ± 2.2 c	326.8 ± 3.2 a	87.4 ± 4.6 a
49	(*Vigna unguiculata* subsp. *Sesquipedalis*)	73.6 ± 1.5 c	100.9 ± 3.0 c	320.0 ± 7.5 ab	87.2 ± 7.4 a
50	(*Vigna unguiculata* subsp. *Sesquipedalis*)	79.9 ± 1.7 b	109.1 ± 3.5 b	331.2 ± 8.4 a	87.9 ± 8.4 a
6	(*Vigna unguiculata*)	38.9 ± 0.1 kl	56.2 ± 0.1 hi	173.4 ± 3.5 e	76.5 ± 1.5 ab

Different letters indicate significant differences at *p* < 0.05.

## Data Availability

All the data supporting the findings of this study are included in this article.

## Supplementary Materials

The following are available online at https://www.mdpi.com/article/10.3390/foods10123118/s1, Figure S1. The collected seeds of Fabaceae species in Thailand: A. *Pisum sativum* L. cv. round pod; B. *Pisum sativum* L. cv. flat pod; C. *Cajanus cajan* (L.) Millsp.; D. *Glycine max* (L.) Merr.; E. *Phaseolus vulgaris* L. cv. white kidney beans; F. *Phaseolus vulgaris* L. cv. red kidney bean; G. *Vigna angularis* (Wild.) Ohwi & H.Ohashi; H. *Vigna mungo* (L.) Hepper; I. *Vigna radiata* (L.) R.Wilczek; J. *Vigna unguiculata* (L.) Walp.; K. *Vigna unguiculata* subsp. *sesquipedalis* (L.) Verdc.; L. *Arachis hypogaea* L.; Bar scale = 1 cm. The photos were taken by D.T. in Thailand; Figure S2: Typical HPLC chromatograms of the different Fabaceae species/subspecies; Table S1. Pearson correlation coefficient linking phytochemicals and antioxidant activity of ethanolic extracts of 50 Fabaceae populations from various floristic regions from Thailand; Table S2. Intraspecific Pearson correlation coefficient linking isoflavone (daidzein and genistein) content and antioxidant activity of ethanolic extracts of 50 Fabaceae populations from various floristic regions from Thailand.
