# Supplementary material for: Characterization of Bioactive Phenolics and Antioxidant Capacity of Edible Bean Extracts of 50 Fabaceae Populations Grown in Thailand
Source: Foods. 2021 Dec 16;10(12):3118. doi: 10.3390/foods10123118 (PMC8700874; doi:10.3390/foods10123118)
Supplement: Supplementary file 1 [file foods-10-03118-s001.zip › foods-1485483-supplementary.pdf]

Supplementary Materials

Characterization of Bioactive Phenolics and Antioxidant Capacity of Edible Bean Extracts of 50 Fabaceae Populations Grown in Thailand

Duangjai Tungmunnithum [1,2,3,*], Samantha Drouet [2], Jose Manuel Lorenzo [4,5] and Christophe Hano [2,3,*]

[1] Department of Pharmaceutical Botany, Faculty of Pharmacy, Mahidol University, Bangkok 10400, Thailand; duangjai.tun@mahidol.ac.th (D.T.)
[2] Laboratoire de Biologie des Ligneux et des Grandes Cultures, INRAE USC1328, Campus Eure et Loir, Orleans University, 28000 Chartres, France; duangjai.tun@mahidol.ac.th (D.T.) ; samantha.drouet@univ-orleans.fr (S.D.) ; hano@univ-orleans.fr (C.H.)
[3] Le Studium Institue for Advanced Studies, 1 Rue Dupanloup, 45000 Orléans, France; duangjai.tun@mahidol.ac.th (D.T.) ; hano@univ-orleans.fr (C.H.)
[4] Centro Tecnológico de la Carne de Galicia, Adva. Galicia n° 4, Parque Tecnológico de Galicia, San Cibrao das Viñas, 32900 Ourense, Spain; jmlorenzo@ceteca.net (J.M.L.)
[5] Área de Tecnología de los Alimentos, Facultad de Ciencias de Ourense, Universidad de Vigo, 32004 Ourense, Spain
* Correspondence: duangjai.tun@mahidol.ac.th (D.T.) ; hano@univ-orleans.fr (C.H.)

Content:

Figure S1. The collected seeds of Fabaceae species in Thailand: A. *Pisum sativum* L. cv. round pod; B. *Pisum sativum* L. cv. flat pod; C. *Cajanus cajan* (L.) Millsp.; D. *Glycine max* (L.) Merr.; E. *Phaseolus vulgaris* L. cv. white kidney beans; F. *Phaseolus vulgaris* L. cv. red kidney bean; G. *Vigna angularis* (Willd.) Ohwi & H.Ohashi; H. *Vigna mungo* (L.) Hepper; I. *Vigna radiata* (L.) R.Wilczek ; J. *Vigna unguiculata* (L.) Walp.; K. *Vigna unguiculata* subsp. *sesquipedalis* (L.) Verdc. ; L. *Arachis hypogaea* L.; Bar scale = 1 cm. The photos were taken by D.T. in Thailand

Figure S2: Typical HPLC chromatograms of the different Fabaceae species/subspecies.

Table S1. Pearson correlation coefficient linking phytochemicals and antioxidant activity of ethanolic extracts of 50 Fabaceae populations from various floristic regions from Thailand.

Table S2. Intraspecific Pearson correlation coefficient linking isoflavone (daidzein and genistein) content and antioxidant activity of ethanolic extracts of 50 Fabaceae populations from various floristic regions from Thailand.

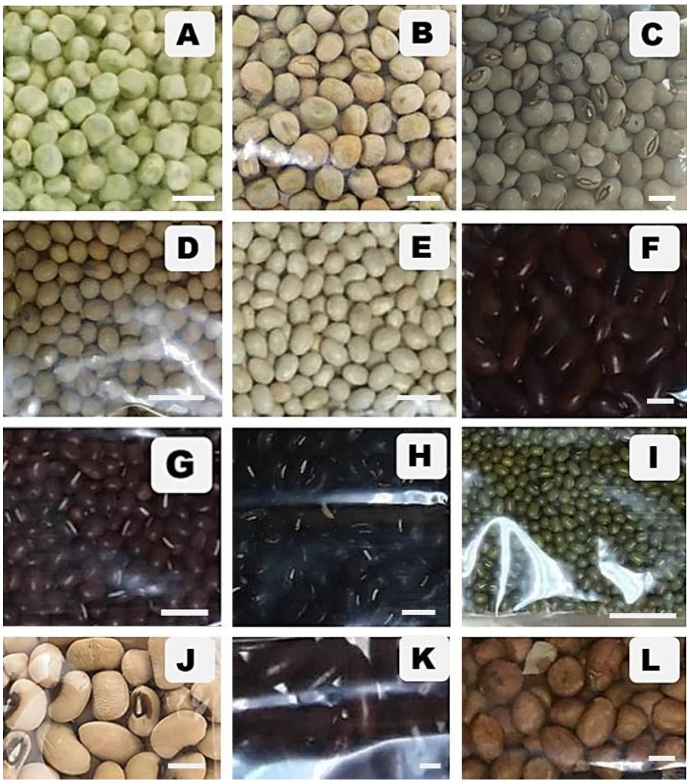

Figure S1. The collected seeds of Fabaceae species in Thailand: A. *Pisum sativum* L. cv. round pod; B. *Pisum sativum* L. cv. flat pod; C. *Cajanus cajan* (L.) Millsp.; D. *Glycine max* (L.) Merr.; E. *Phaseolus vulgaris* L. cv. white kidney beans; F. *Phaseolus vulgaris* L. cv. red kidney bean; G. *Vigna angularis* (Willd.) Ohwi & H.Ohashi; H. *Vigna mungo* (L.) Hepper; I. *Vigna radiata* (L.) R.Wilczek ; J. *Vigna unguiculata* (L.) Walp.; K. *Vigna unguiculata* subsp. *sesquipedalis* (L.) Verdc. ; L. *Arachis hypogaea* L.; Bar scale = 1 cm. The photos were taken by D.T. in Thailand

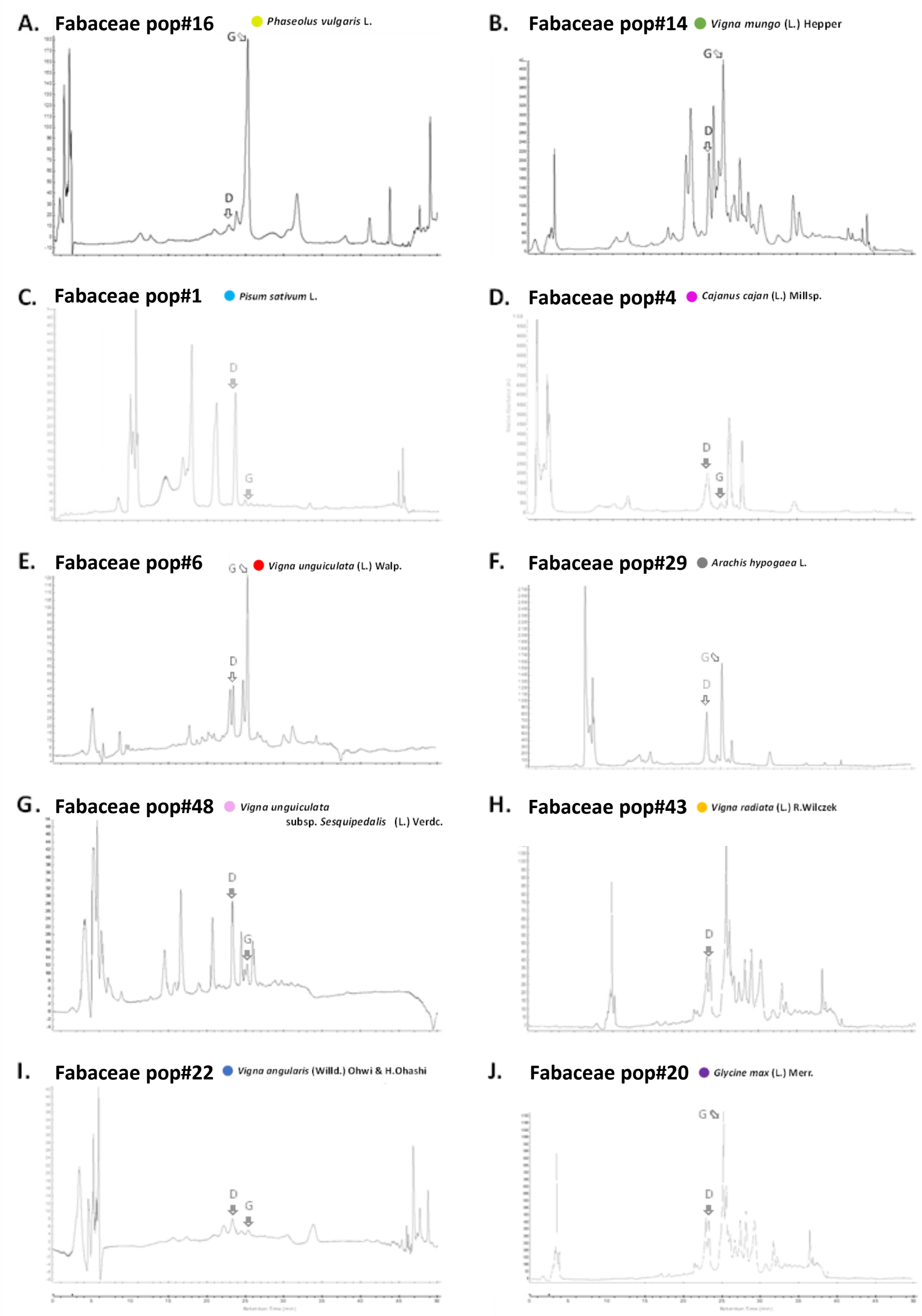

Figure S2: Typical HPLC chromatograms of the different Fabaceae species/subspecies.

Table S1. Pearson correlation coefficient linking phytochemicals and antioxidant activity of ethanolic extracts of 50 Fabaceae populations from various floristic regions from Thailand.

	TPC	TFC	TAC	DAD	GEN	DPPH	ABTS	FRAP	CAA
**TPC**									
**TFC**	0.748 ***								
**TAC**	0.691 ***	0.929 ***							
**DAD**	0.088 ns	-0.161 ns	-0.046 ns						
**GEN**	0.085 ns	-0.165 ns	-0.063 ns	0.983 ***					
**DPPH**	0.389 **	0.625 ***	0.614 ***	0.145 ns	0.125 ns				
**ABTS**	0.381 **	0.625 ***	0.631 ***	0.163 ns	0.143 ns	0.947 *			
**FRAP**	0.764 ***	0.733 ***	0.648 ***	-0.241 ns	-0.246 ns	0.342 ***	0.340 ***		
**CAA**	0.650 ***	0.650 ***	0.606 ***	-0.065 ns	-0.077 ns	0.514 ***	0.518 ***	0.907 ***	

*** significant $p < 0.001$; ** significant $p < 0.01$; * significant $p < 0.05$; ns: non-significant $p > 0.05$. TPC: total phenolic content; TFC: total flavonoid content; TAC: total anthocyanin content; DAD: dadzein content; GEN: genistein content; DPPH: in vitro antioxidant DPPH assay; ABTS: in vitro antioxidant ABTS assay; FRAP: in vitro antioxidant FRAP assay; CAA: cellular antioxidant assay.

Table S2. Intraspecific Pearson correlation coefficient linking isoflavone (daidzein and genistein) content and antioxidant activity of ethanolic extracts of 50 Fabaceae populations from various floristic regions from Thailand.

	DPPH	ABTS	FRAP	CAA
		***Arachis hypogaea* L.**		
**DAD**	-0.569 ns	-0.568 ns	0.983 **	0.981 **
**GEN**	-0.635 ns	-0.635 ns	0.982 **	0.998 ***
		***Cajanus cajan* (L.) Millsp.**		
**DAD**	0.999 *	0.99987**	0.999 **	0.999 **
**GEN**	0.999 *	0.99823*	0.997 *	0.997 *
		***Glycine max* (L.) Merr.**		
**DAD**	-0.172 ns	-0.172 ns	0.568 ns	0.362 ns
**GEN**	-0.543 ns	-0.543 ns	0.461 ns	0.084 ns
		***Phaseolus vulgaris* L. cv. red kidney bean**		
**DAD**	0.015 ns	0.015 ns	0.031 ns	0.105 ns
**GEN**	0.057 ns	0.057 ns	0.108 ns	0.177 ns
		***Pisum sativum* L.**		
**DAD**	0.999 ***	0.999 ***	0.999 ***	0.999 ***
**GEN**	0.999 *	0.999 *	0.999 ***	0.999 ***
		***Vigna angularis* (Willd.) Ohwi & H.Ohashi**		
**DAD**	0.887 *	0.887 *	0.580 ns	0.846 *
**GEN**	0.911 *	0.911 *	0.671 ns	0.898 *
		**Vigna mungo (L.) Hepper**		
**DAD**	0.373 ns	0.373 ns	0.963 **	0.941 **
**GEN**	0.489 ns	0.489 ns	0.983 ***	0.976 ***
		***Vigna radiata* (L.) R.Wilczek**		
**DAD**	0.458 ns	0.458 ns	0.763 ns	0.773 ns
**GEN**	0.218 ns	0.218 ns	0.518 ns	0.516 ns
		***Vigna unguiculata* (L.) Walp.**		
**DAD**	0.998 *	-0.999 *	0.999 *	-0.995 ns
**GEN**	0.979 ns	-0.985 ns	0.982 ns	-0.971 ns
		***Vigna unguiculata* subsp. *Sesquipedalis* (L.) Verdc.**		
**DAD**	0.733 ns	0.733 ns	0.083 ns	0.691 ns
**GEN**	0.911 ns	0.911 ns	0.342 ns	0.884 ns

*** significant $p < 0.001$; ** significant $p < 0.01$; * significant $p < 0.05$; ns: non-significant $p > 0.05$. DAD: dadzein content; GEN: genistein content; DPPH: in vitro antioxidant DPPH assay; ABTS: in vitro antioxidant ABTS assay; FRAP: in vitro antioxidant FRAP assay; CAA: cellular antioxidant assay.
